# GPT Fusion: Integrating Convolutional Neural Networks with GPT-5.5 for Melanoma Diagnosis

**DOI:** 10.21203/rs.3.rs-10500601/v1

**Published:** 2026-07-28

**Authors:** Katie L. Frederickson, Dong Li, Samuel E. Adunyah, Qingguo Wang

**Affiliations:** Meharry Medical College; University of Chicago; Meharry Medical College; Meharry Medical College

**Keywords:** melanoma diagnosis, dermoscopy, large language model, convolutional neural network, ChatGPT, GPT-5.5

## Abstract

**Background:**

Malignant melanoma (MM) is the most aggressive form of metastatic skin cancer, for which early detection is critical to reducing morbidity and mortality while improving patient outcomes. Recent advances in multimodal large language models (LLMs) have demonstrated considerable potential for melanoma detection and clinical decision support. However, their diagnostic accuracy remains inferior to that of specialized convolutional neural networks (CNNs). Whether LLMs can effectively integrate predictions from specialized CNN models to improve melanoma diagnosis has not been systematically investigated.

**Methods:**

We developed a hybrid diagnostic approach, termed GPT Fusion, in which GPT-5.5 serves as a reasoning-based fusion framework rather than an image classifier. Instead of directly analyzing lesion images, GPT-5.5 received structured outputs from two independently developed CNN models: a multimodal ResNet-50 model trained on the MILK10K dataset for multiclass skin lesion classification and the first-place 90-model SIIM-ISIC ensemble optimized for melanoma detection. GPT-5.5 integrated these complementary predictions to generate the final diagnosis. The framework was evaluated using the publicly available Derm7pt dataset. Performance was assessed for melanoma prediction (melanoma vs. non-melanoma), malignancy prediction (malignant vs. benign lesions), primary diagnosis, and top-3 differential diagnosis.

**Results:**

GPT Fusion substantially outperformed image-based GPT-5.5 across all evaluation tasks. For melanoma prediction, accuracy improved from 63.3% to 86.9%, while ROC AUC increased from 0.746 to 0.923 and PR AUC from 0.545 to 0.853. For malignancy prediction, GPT Fusion not only exceeded the performance of image-based GPT-5.5 but also surpassed the specialized SIIM-ISIC ensemble in sensitivity (83.9%), F1 score (73.6%), ROC AUC (0.894), and PR AUC (0.823), while maintaining comparable overall accuracy. GPT Fusion further improved primary diagnosis accuracy and achieved the highest top-3 differential diagnosis accuracy (89.0%).

**Conclusions:**

These findings demonstrate that LLMs can provide greater clinical value as reasoning-based integrators of specialized AI systems than as standalone image classifiers. By combining the complementary strengths of specialized CNN models, GPT-5.5 substantially improved diagnostic performance while providing interpretable reasoning. This hybrid strategy offers a promising direction for developing more accurate, transparent, and clinically useful AI systems for melanoma diagnosis.

## 1. INTRODUCTION

Malignant melanoma (MM) is a high-risk neoplasm arising from melanocytes, the pigment-producing cells of the skin. It is the most aggressive form of skin cancer and has a high propensity for metastasis to regional and distant organs. The American Cancer Society (ACS) estimates approximately 112,000 new MM cases will be diagnosed in 2026, including approximately 65,400 cases in men and 46,600 in women [1]. An estimated 8,510 deaths are expected in the same year.

Early identification of melanoma is strongly associated with improved patient survival. However, accurate diagnosis remains challenging because early melanoma lesions often resemble benign pigmented lesions such as nevi or seborrheic keratoses. Diagnostic difficulty is further compounded by variations in skin pigmentation and lesion morphology across populations. To address these challenges, considerable research has focused on applying artificial intelligence (AI), in particular machine learning (ML) and deep learning (DL), to melanoma detection. Among DL approaches, convolutional neural networks (CNNs), deep learning models that automatically learn hierarchical visual features from medical images through successive convolutional layers, have become the dominant framework for image classification and lesion recognition. CNN-based architectures, such as Inception-v4, ResNet, DenseNet, and EfficientNet, have achieved dermatologist-level performance in landmark studies [2–5]. These advances have also translated into clinical practice, with AI-assisted tools such as FotoFinder’s Moleanalyzer-Pro and DermaSensor’s spectroscopy-based device supporting dermoscopic assessment [6, 7]. Nevertheless, despite their high diagnostic accuracy, these specialized CNN models function primarily as black-box classifiers, providing limited interpretability, clinical reasoning, and interactive decision support.

Timely melanoma detection is further complicated by limited access to dermatologic care in distant, rural areas. In the United States, dermatology residency programs offer only 574 positions annually, contributing to workforce constraints. A recent study reported a mean wait time of 50 days for a new patient dermatology appointment [8]. These barriers to timely dermatologic evaluation may contribute to delayed detection, disease progression, and increased melanoma-related morbidity and mortality. Therefore, AI-based diagnostic systems that expand access to melanoma screening and support of timely clinical diagnosis are urgently required.

Recent advances in large language models (LLMs) have introduced a new paradigm for medical AI. Unlike conventional CNNs, modern multimodal LLMs can integrate real-time visual, auditory, and textual information to generate diagnoses and natural-language explanations. Beginning with GPT-4 Vision (GPT-4V), LLMs have incorporated the ability to analyze user-uploaded medical images [9–11]. The recently released GPT-5 further improved diagnostic reasoning, robustness, and resistance to hallucinations across heterogeneous clinical inputs [9, 12, 13]. These developments position LLMs as promising tools for interactive clinical decision support that extend beyond image classification alone [14].

Accordingly, numerous studies have investigated the use of LLMs for melanoma detection. Shifai et al. evaluated GPT-4V using images from the International Skin Imaging Collaboration (ISIC) Archive [15]. Similarly, Liu et al. compared GPT-4V with Claude 3 Opus on the ISIC dataset [16]. Using the Human Against Machine with 10,000 training images (HAM10K) dataset, Sattler et al. evaluated GPT-4 Turbo (GPT-4T) and ChatGPT-4 Omni (GPT-4o), reporting variable diagnostic sensitivity, specificity, and accuracy [17]. Boostani et al. compared ChatGPT-4o and Gemini 2.0 Flash and found that their performance differed across clinical and dermoscopic images, and that combining the two models improved detection sensitivity [18]. More recently, Wang et al. evaluated GPT-5 comprehensively across the ISIC, HAM10K, and MILK10K datasets, demonstrating moderate performance in primary diagnosis and malignancy classification but substantially higher top-3 differential diagnosis accuracy [13, 19].

Collectively, these findings suggest that although current multimodal LLMs demonstrate considerable diagnostic potential, they remain insufficiently reliable as standalone melanoma classifiers. Conversely, specialized CNN models generally achieve higher diagnostic accuracy but lack the reasoning, contextual understanding, and interactive communication capabilities of LLMs. These complementary strengths motivate integrating the two approaches to develop a unified diagnostic framework, in which CNNs provide accurate image-based predictions, while LLMs synthesize CNN outputs, reconcile potentially conflicting predictions, incorporate relevant clinical information, and generate clinically meaningful diagnostic assessments through natural-language interaction with users.

In this study, we assessed a hybrid diagnostic strategy, termed GPT Fusion, in which GPT-5.5 functions as a reasoning-based meta-classifier that integrates the outputs of two specialized CNN systems: (i) a ResNet-50 model trained on the MILK10K dataset for broad skin lesion classification using paired clinical and dermoscopic images [20], and (ii) the first-place ensemble from the SIIM-ISIC Melanoma Classification Challenge, comprising multiple CNN models optimized specifically for binary melanoma detection [21]. Rather than classifying lesion images directly, GPT-5.5 in the framework serves as a reasoning-based diagnostic integrator by synthesizing the structured outputs of these specialized models to generate a consensus diagnosis and malignancy assessment. To our knowledge, this study represents the first systematic evaluation of an LLM that integrates state-of-the-art CNNs for melanoma diagnosis.

## 2. MATERIALS AND METHODS

**Figure 1:**
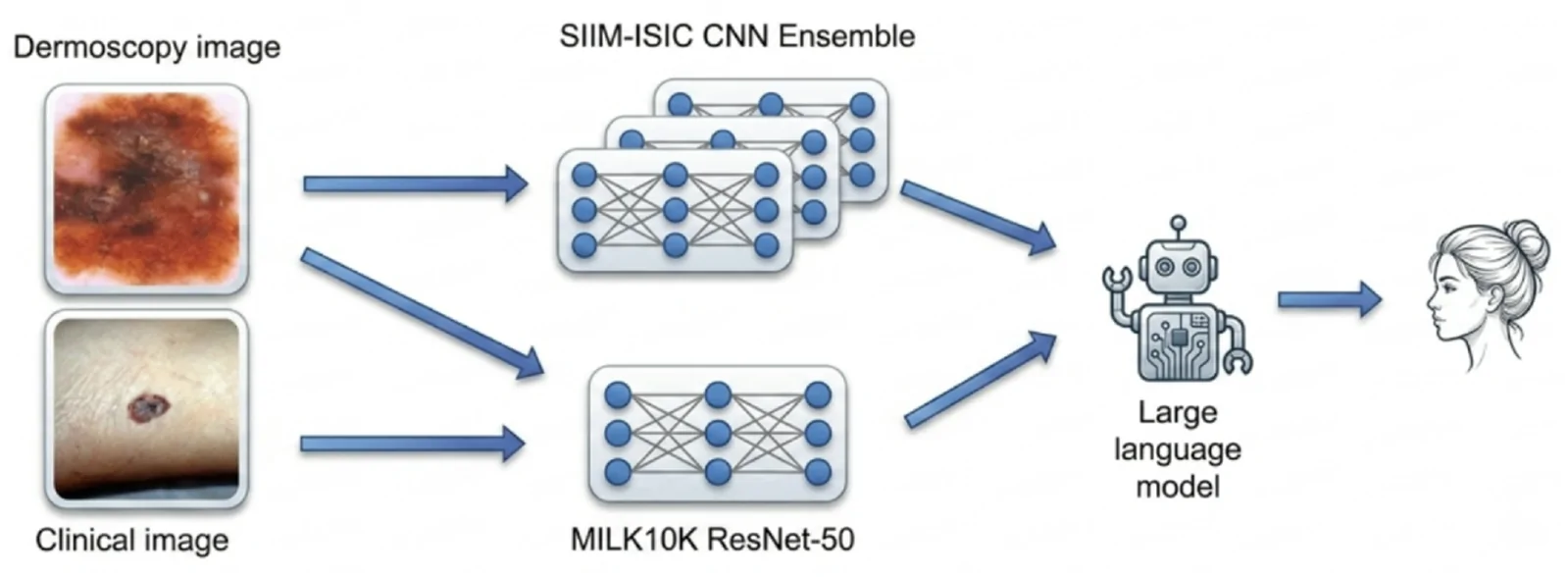
Overview of the proposed GPT Fusion framework. Dermoscopic images are analyzed by the SIIM-ISIC 90-model CNN ensemble, while paired dermoscopic and clinical images are analyzed by the ResNet-50 model trained on MILK10K. Rather than interpreting images directly, the LLM GPT-5.5 integrates the structured prediction outputs from both CNN models to generate a consensus diagnosis, differential diagnosis, melanoma probability, malignancy probability, and diagnostic rationale.

This study investigated whether an LLM can improve melanoma diagnosis by integrating the complementary strengths of specialized CNN models. Figure 1 provides an overview of the proposed GPT Fusion framework and the study workflow. Specifically, we compared four diagnostic paradigms: image-based GPT-5.5, a multimodal ResNet-50 model, the SIIM-ISIC CNN ensemble, and the proposed GPT Fusion framework, using the publicly available Derm7pt dataset [22]. Dermoscopy, the most widely adopted and clinically validated imaging modality for melanoma detection [23], served as the primary imaging modality throughout this study.

### 2.1 Derm7pt Dataset

All evaluations were performed using the Derm7pt dataset [22], which contains 1011 pairs of clinical close-up and dermoscopic images with expert-confirmed histopathological or clinical reference diagnoses and comprehensive clinical metadata. The dataset also includes annotations based on the seven-point checklist, facilitating the development and evaluation of computer-aided diagnostic systems. Derm7pt was selected as an independent external benchmark because neither the widely used ISIC Archive nor the MILK10K datasets were suitable for evaluation, as both had been used to train the CNN models assessed in this study.

The reference diagnosis provided in the metadata served as the ground truth for all performance evaluations. Because the MILK10K ResNet-50 model was trained using a different diagnostic taxonomy and the SIIM-ISIC CNN ensemble was designed exclusively for binary melanoma classification, diagnostic labels were harmonized before comparative analyses, and all diagnoses were mapped to the standardized diagnostic categories used in Derm7pt.

### 2.2 Image-Based CNN Models

#### ResNet-50.

Because the MILK10K investigators did not publicly release pretrained model weights, we reproduced their ResNet-50 model using the authors’ publicly available code and dataset without modification [20]. The model was trained on MILK10K following the original training protocol, and its performance was verified on the MILK10K test set (Appendix Table A1). The validated model was then applied directly to the Derm7pt dataset without further fine-tuning or parameter optimization. The multimodal ResNet-50 model accepts paired clinical close-up and dermoscopic images and predicts 48 fine-grained skin lesion diagnoses together with calibrated class probabilities. To facilitate clinical interpretation, the MILK10K investigators grouped these 48 diagnoses into 11 broader diagnostic categories, with each detailed diagnosis assigned to a single category. For comparative evaluation in this study, the ResNet-50 predictions were further mapped to the harmonized Derm7pt diagnostic categories.

#### SIIM-ISIC CNN Ensemble.

The publicly released model checkpoints from the first-place solution of the SIIM-ISIC Melanoma Classification Challenge were downloaded directly from the Kaggle repository and evaluated without retraining or modification (Appendix Table A2) [21]. The winning solution employed 18 distinct model configurations, primarily based on EfficientNet and ResNeXt architectures operating at multiple image resolutions. Each configuration was trained using 5-fold cross-validation, resulting in an ensemble of 90 trained model checkpoints (hereafter referred to as the SIIM-90 Ensemble). Each checkpoint generated a continuous probability of melanoma for each lesion. Predictions from the checkpoints were combined using two ensemble strategies: (i) a probability ensemble, in which melanoma probabilities were averaged across all checkpoints, and (ii) the official SIIM rank ensemble, which aggregates normalized prediction rankings across model configurations.

### 2.3 Reasoning-Based Diagnostic Fusion

To integrate the complementary strengths of CNNs and LLMs, we have LLMs (GPT-5.5, a leading LLM, was used in this study) serve as a reasoning-based meta-classifier rather than an image classifier. Unlike the image-based assessment, GPT-5.5 was not provided with either the clinical close-up or dermoscopic images. Instead, it received only the structured textual and numerical outputs generated by the ResNet-50 and SIIM-ISIC ensemble models, including the primary diagnosis, top-3 differential diagnoses, class probabilities, melanoma probability, malignancy probability, and threshold-adjusted binary predictions.

The standardized prompt explicitly informed GPT-5.5 of the complementary roles of the two AI models. Specifically, the SIIM-ISIC ensemble model was described as optimized for melanoma detection and therefore should receive greater weight when estimating melanoma probability, whereas the ResNet-50 model was described as providing broader multiclass diagnostic information and should primarily contribute to differential diagnosis and the assessment of non-melanoma malignancies. GPT-5.5 was instructed to integrate these complementary predictions to generate a consensus diagnosis.

### 2.4 Diagnostic Tasks

Two binary classification tasks were evaluated. Melanoma prediction assessed the ability to distinguish melanoma from all other skin lesions, including both benign lesions and non-melanoma malignancies. In contrast, malignancy prediction assessed the ability to distinguish all malignant skin lesions (e.g., melanoma, basal cell carcinoma, squamous cell carcinoma, and other malignant tumors) from benign lesions. Thus, melanoma prediction assesses performance on a disease-specific task, whereas malignancy prediction reflects the broader clinical objective of identifying lesions that require prompt diagnostic evaluation and treatment.

In addition to the two binary classification tasks, diagnostic performance was evaluated using top-1 primary and top-3 differential diagnosis accuracy after mapping lesion diagnoses to standardized diagnostic categories. Top-1 primary diagnosis accuracy represents the percentage of lesions for which the model’s first (highest-confidence) diagnosis is correct. Top-3 differential diagnosis accuracy represents the percentage of lesions for which the correct diagnosis is included among the model’s three highest-ranked candidate diagnoses. Whereas top-1 accuracy evaluates the ability to identify the single best diagnosis, top-3 accuracy reflects the ability to generate an appropriate differential diagnosis, a process that closely resembles clinical practice in which physicians consider several plausible diagnoses before confirming the final diagnosis.

### 2.5 Performance Evaluation

For these diagnostic tasks, performance was evaluated using accuracy, sensitivity, specificity, positive predictive value (PPV), negative predictive value (NPV), F1 score, receiver operating characteristic area under the curve (ROC AUC), and precision-recall area under the curve (PR AUC). For the ResNet-50 and SIIM-ISIC models, optimal decision thresholds were determined by maximizing the Youden index (*J*), *J = Sensitivity + Specificity − 1*, which identifies the threshold that jointly maximizes sensitivity and specificity. The optimized thresholds were subsequently fixed and applied throughout all LLM fusion experiments.

### 2.6 Implementation

The complete evaluation pipeline was implemented in Python. Model outputs were parsed, normalized, and benchmarked against the Derm7pt reference standards. Continuous prediction scores were utilized to generate ROC and precision-recall curves, while binary metrics were evaluated using either the optimized operating thresholds or model-generated binary decisions, as appropriate. The source code developed for this study, including evaluation scripts for the ResNet-50 model, the SIIM-ISIC ensemble, and the LLM-based diagnostic fusion framework, will be made publicly available at https://github.com/qwangmsk/Melanoma-Detect.

## 3. RESULTS

### 3.1 Melanoma Prediction

**Figure 2:**
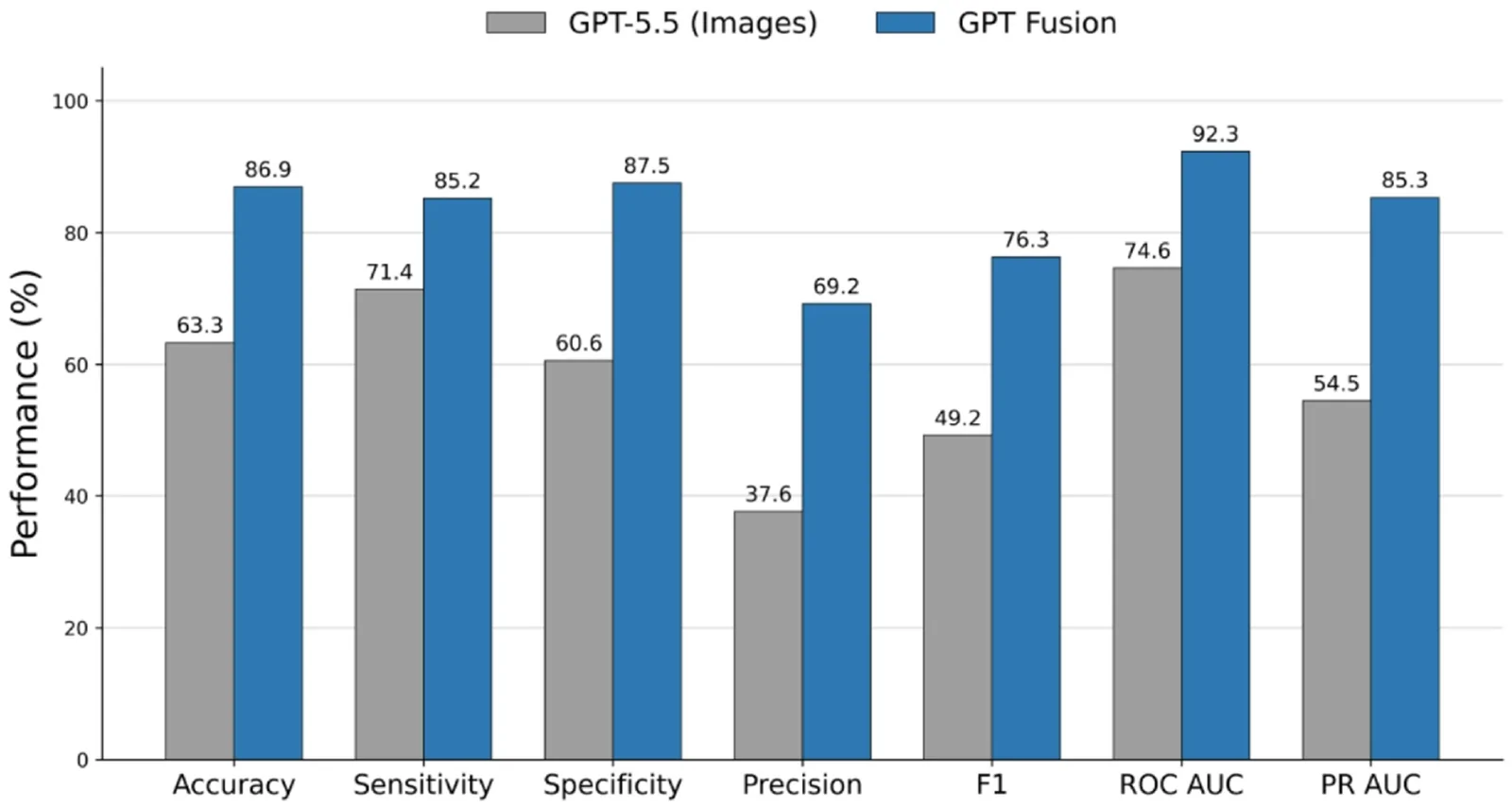
Melanoma prediction performance: image-based GPT-5.5 vs GPT Fusion

For each skin lesion in Derm7pt, GPT Fusion generated a melanoma prediction by integrating the outputs of the ResNet-50 and SIIM-ISIC ensemble models. Its performance, together with those of the individual models and image-based GPT-5.5, is summarized in Table 1 and Fig. 2.

The SIIM-90 ensemble and GPT Fusion substantially outperformed image-based GPT-5.5 and ResNet-50 across most performance measures. GPT Fusion achieved the highest sensitivity, ROC AUC, and PR AUC, at 85.2%, 0.923, and 0.853, respectively. Its overall performance was comparable to that of the SIIM-90 ensemble, which showed slightly higher accuracy, specificity, precision, and F1 score. Relative to image-based GPT-5.5, GPT Fusion improved accuracy from 63.3% to 86.9%, specificity from 60.6% to 87.5%, precision from 37.6% to 69.2%, and F1 score from 49.2% to 76.3%. These findings indicate that reasoning over complementary CNN outputs substantially enhanced melanoma discrimination compared with direct image-based LLM assessment, i.e., GPT-5.5 (Images) in Table 1 and Fig. 2.

The comparatively modest performance of the ResNet-50 model on the Derm7pt dataset likely reflects both its limited representational capacity and limited domain generalization. ResNet-50 is a relatively early CNN architecture that is generally less powerful than modern ensemble-based and transformer-based models for skin lesion classification. Moreover, the model was trained exclusively on the MILK10K dataset, which contains paired dermoscopic and clinical close-up images acquired using standardized imaging protocols. The clinical images in Derm7pt differ substantially in image composition, magnification, field of view, illumination, and lesion presentation. Whereas the MILK10K clinical images were collected specifically to complement dermoscopic assessment, the Derm7pt clinical photographs were acquired under different clinical conditions and exhibit greater variability in lesion size, surrounding skin, and photographic quality. Consequently, the image distribution encountered during external evaluation differs markedly from that seen during training, resulting in domain shift that further degrades performance. Together, these findings suggest that robust multimodal skin lesion classification requires not only more expressive model architectures but also training on more diverse datasets to improve generalization across clinical settings.

### 3.2 Malignancy Prediction

**Figure 3:**
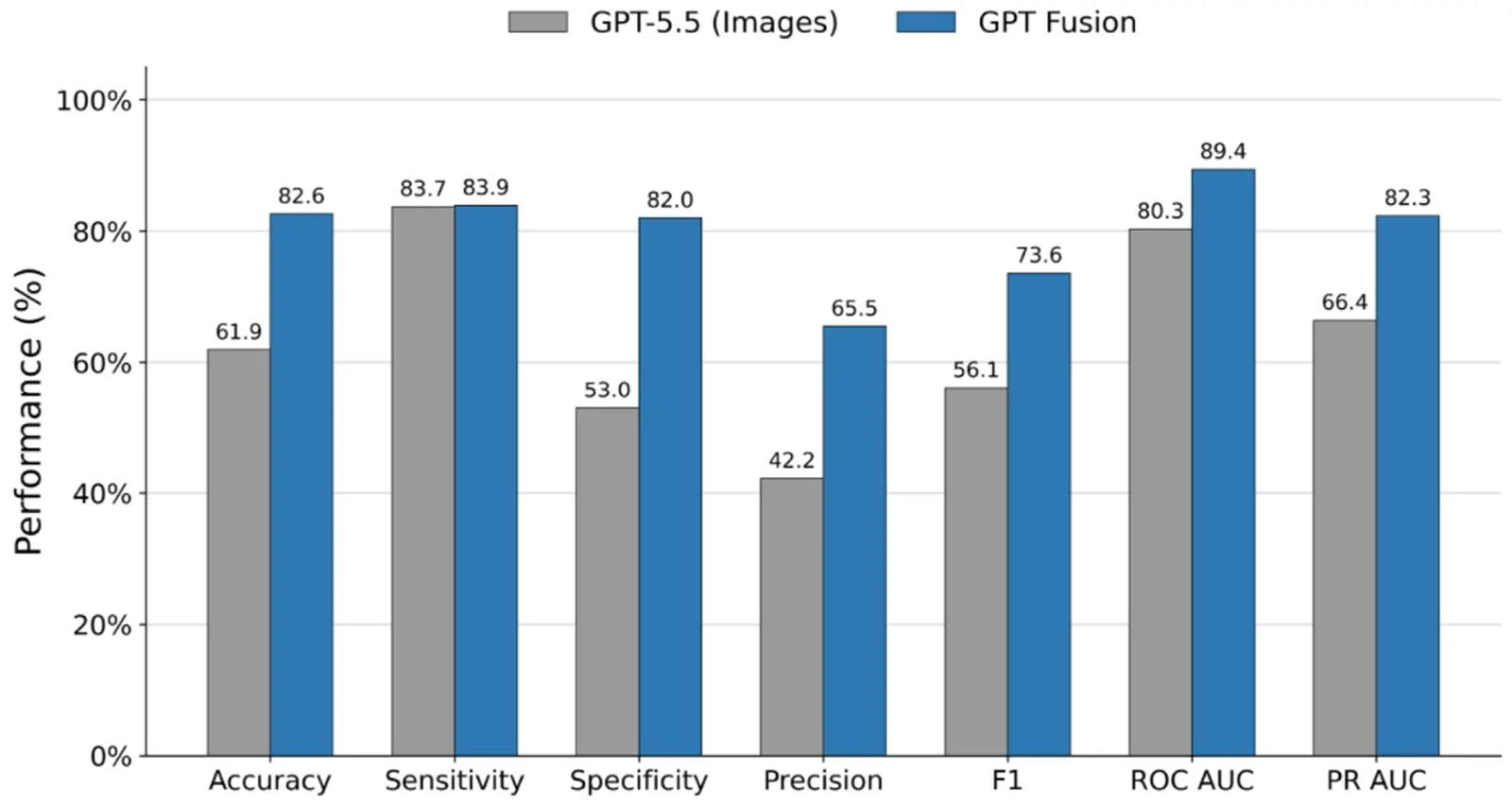
Malignancy Prediction Performance: Image-Based GPT-5.5 vs GPT Fusion

Unlike melanoma prediction, which focuses exclusively on melanoma detection, malignancy prediction evaluates the ability to identify all malignant skin lesions. Consequently, this task provides a broader assessment of the clinical utility of the proposed framework. For each skin lesion, the fusion framework generated a binary malignancy prediction by integrating the outputs of the ResNet-50 and SIIM-ISIC ensemble models. The resulting performance, together with those of the individual models and image-based GPT-5.5, is summarized in Table 2 and Fig. 3.

The SIIM-90 ensemble and GPT Fusion substantially outperformed image-based GPT-5.5 and ResNet-50 for malignancy prediction. GPT Fusion achieved the highest sensitivity (83.9%), F1 score (73.6%), ROC AUC (0.894), and PR AUC (0.823), indicating the best overall discrimination between malignant and benign lesions. In comparison, the SIIM-90 ensemble demonstrated slightly higher accuracy (83.5%), specificity (86.5%), and precision (69.8%). As shown in Fig. 3, relative to image-based GPT-5.5, GPT Fusion improved accuracy from 61.9% to 82.6%, specificity from 53.0% to 82.0%, precision from 42.2% to 65.5%, F1 score from 56.1% to 73.6%, ROC AUC from 0.803 to 0.894, and PR AUC from 0.664 to 0.823, while maintaining comparable sensitivity (83.7% vs. 83.9%). These results demonstrate that reasoning-based integration of complementary CNN predictions substantially improves malignancy discrimination compared with direct image-based LLM assessment while achieving performance comparable to, or exceeding, that of the specialized SIIM-90 ensemble.

Although the MILK10K ResNet-50 model demonstrated lower standalone performance than the SIIM-90 ensemble for both melanoma and malignancy prediction on Derm7pt, it remained an important contributor to the proposed fusion framework. The SIIM-90 ensemble, developed specifically for melanoma detection, provides highly accurate melanoma probabilities but relatively limited information about other malignant skin lesions. In contrast, the MILK10K ResNet-50 model generates multiclass predictions spanning a broader spectrum of benign and malignant lesion types. By reasoning over these complementary outputs rather than relying on either model alone, GPT-5.5 was able to partially compensate for the limitations of the individual CNN models.

This complementary effect was most evident in malignancy prediction. Although GPT Fusion and the SIIM-90 ensemble achieved comparable performance for melanoma prediction, GPT Fusion surpassed the SIIM-90 ensemble in sensitivity, F1 score, ROC AUC, and PR AUC for distinguishing malignant from benign lesions, indicating that reasoning-based integration is particularly advantageous for broader malignancy discrimination, where diagnostic decisions require distinguishing among multiple malignant and benign lesion categories rather than identifying melanoma alone.

### 3.3 Diagnostic category classification performance

Table 3 summarizes diagnostic category classification performance for the evaluated models. GPT Fusion achieved the highest top-1 primary diagnosis accuracy (69.8%), representing a substantial improvement over both image-based GPT-5.5 (42.6%) and the ResNet-50 model (53.6%). For the top-3 differential diagnosis, GPT Fusion also achieved the best performance (89.0%), although the improvement over image-based GPT-5.5 (88.8%) was modest. In contrast, the ResNet-50 model yielded a lower top-3 diagnosis accuracy (84.7%). These findings suggest that integrating the complementary outputs of the ResNet-50 and SIIM-ISIC models enabled GPT-5.5 to substantially improve its primary diagnostic classification while maintaining excellent differential diagnostic performance.

The SIIM-ISIC ensemble was excluded from this analysis because it was developed specifically for binary melanoma classification. Unlike GPT-5.5 and the ResNet-50 model, it does not produce primary diagnoses or top-3 differential diagnoses and therefore cannot be evaluated using diagnostic category classification metrics.

## 4. DISCUSSION

AI has transformed melanoma diagnosis over the past decade. Specialized CNN models have achieved dermatologist-level performance on benchmark datasets and have been incorporated into clinical decision-support systems. More recently, multimodal LLMs have demonstrated the potential to interpret medical images, provide natural-language explanations, and offer interactive clinical support. These two technologies have complementary strengths: CNNs excel at image pattern recognition but generally lack interpretability, whereas LLMs offer reasoning capability and interactive decision support but currently remain less accurate than specialized CNNs for melanoma diagnosis. This study investigated whether these complementary strengths could be combined through a reasoning-based diagnostic fusion strategy.

Our results demonstrated that GPT Fusion consistently outperformed image-based GPT-5.5 across multiple clinically relevant diagnostic tasks. Compared with direct image interpretation by GPT-5.5, the proposed framework substantially improved melanoma prediction, malignancy discrimination, and diagnostic category classification. These findings suggest that LLMs contribute more effectively to melanoma diagnoses by reasoning over structured outputs generated by specialized diagnostic models than by directly interpreting dermoscopic images. Rather than functioning as standalone image classifiers, LLMs may be better suited to serve as intelligent diagnostic integrators capable of synthesizing complementary evidence from multiple AI systems.

One notable finding was that the multimodal MILK10K ResNet-50 model, despite its relatively modest standalone performance on Derm7pt, remained an important contributor to the GPT Fusion framework. Its lower performance likely reflects both the limited representational capacity of the ResNet-50 architecture and limited domain generalization, as the model was trained exclusively on the standardized MILK10K dataset. Nevertheless, ResNet-50 provided broad multiclass predictions that complemented the highly accurate melanoma probabilities generated by the SIIM-ISIC ensemble. By reasoning over these complementary outputs, GPT-5.5 was able to partially compensate for the limitations of the individual CNN models.

The advantages of this reasoning-based integration were most evident for malignancy prediction. Whereas melanoma prediction focuses on identifying a single cancer subtype, malignancy prediction requires distinguishing a broader spectrum of malignant lesions from benign lesions. Accordingly, GPT Fusion achieved melanoma prediction performance comparable to that of the SIIM-ISIC ensemble while surpassing it in sensitivity, F1 score, ROC AUC, and PR AUC for overall malignancy discrimination. This improvement is consistent with the complementary roles of the two CNN models: the SIIM-ISIC ensemble was optimized specifically for melanoma detection, whereas the MILK10K ResNet-50 model was designed for broader multiclass skin lesion classification. By integrating these complementary sources of evidence, GPT-5.5 preserved the strong melanoma discrimination of the SIIM-ISIC ensemble while incorporating additional diagnostic information relevant to other malignant lesion types. These results suggest that reasoning-based integration is particularly advantageous for clinical decision-making tasks that require discrimination among multiple malignant and benign skin lesions, rather than for melanoma detection alone.

The tested framework, GPT Fusion, differs fundamentally from conventional ensemble learning. Traditional ensemble methods combine model outputs mathematically through probability averaging, majority voting, or weighted combinations. Although these approaches often improve predictive performance, they cannot explicitly reason about conflicting predictions, exploit complementary diagnostic information, or generate interpretable explanations for their conclusions. In contrast, GPT Fusion integrates structured outputs from multiple specialized models and produces consensus diagnoses together with diagnostic rationales. Such reasoning-based integration may improve transparency and facilitate clinician trust, particularly in educational and clinical decision-support settings where understanding the basis for a prediction is as important as the prediction itself.

Several limitations should be acknowledged. First, the proposed framework was evaluated using a single external dataset; validation across larger, more diverse clinical cohorts is needed to establish its generalizability. Second, only one LLM and two CNN models were investigated. Future studies should evaluate whether similar improvements can be achieved using alternative LLMs and additional specialized dermatologic AI models. Third, although GPT Fusion generated interpretable diagnostic rationales, the present study focused on diagnostic performance rather than the clinical usefulness or factual reliability of these explanations. Future work should also investigate prospective clinical validation, more sophisticated multi-agent architectures, and seamless integration into dermatologist workflows.

## 5. CONCLUSIONS

This study assessed GPT Fusion, a hybrid melanoma diagnosis framework that uses GPT-5.5 as a reasoning-based meta-classifier to integrate predictions from specialized CNN models rather than directly interpreting lesion images. By combining the complementary strengths of a melanoma-specialized SIIM-ISIC ensemble (trained on the ISIC Archive) and a multimodal ResNet-50 model (trained on MILK10K), GPT Fusion substantially improved diagnostic performance over image-based GPT-5.5 and achieved performance comparable to or better than the specialized CNN models across several clinically relevant tasks. These findings suggest that the greatest clinical value of LLMs in dermatologic diagnosis may lie not in replacing specialized image-analysis models, but in orchestrating and interpreting their outputs through reasoning-based integration. More broadly, this study demonstrates a promising strategy for developing transparent, accurate, and clinically useful hybrid AI systems for melanoma diagnosis and provides a foundation for extending reasoning-based AI integration to other areas of medical imaging and clinical decision support.

## Supplementary Material

Supplementary Files

This is a list of supplementary files associated with this preprint. Click to download.

- APPENDIX.docx

## Figures and Tables

**Table 1:** Performance comparison of four AI approaches for melanoma prediction on the Derm7pt dataset.

Model	Accuracy	Sensitivity	Specificity	Precision	F1	ROC AUC	PR AUC
GPT-5.5 (Images)	63.3	71.4	60.6	37.6	49.2	0.746	0.545
ResNet-50	71.0	67.1	72.3	44.6	53.6	0.742	0.493
SIIM-90 Ensemble	87.2	84.9	88.0	70.2	76.8	0.922	0.849
GPT Fusion	86.9	85.2	87.5	69.2	76.3	0.923	0.853

**Table 2:** Performance comparison of four AI approaches for malignancy prediction on the Derm7pt dataset.

Model	Accuracy	Sensitivity	Specificity	Precision	F1	ROC AUC	PR AUC
GPT-5.5 (Images)	61.9	83.7	53.0	42.2	56.1	0.803	0.664
ResNet-50	70.9	73.5	69.9	50.0	59.5	0.760	0.541
SIIM-90 Ensemble	83.5	76.2	86.5	69.8	72.8	0.857	0.809
GPT Fusion	82.6	83.9	82.0	65.5	73.6	0.894	0.823

**Table 3:** Performance comparison of three AI approaches for diagnostic category classification performance on the Derm7pt dataset.

Model	Input	Top-1 Primary Diagnosis Accuracy	Top-3 Differential Diagnosis
GPT-5.5 (Images)	Images	42.6%	88.8%
ResNet-50	Clinical + Dermoscopy	53.6%	84.7%
GPT Fusion	ResNet + SIIM outputs	69.8%	89.0%

## Data Availability

Derm7pt data are open for research use, and do not include proprietary or patient-identifiable data. All Derm7pt dermoscopic and clinical images, along with metadata, analyzed in this study are publicly available and can be assessed at https://github.com/jeremykawahara/derm7pt. Additionally, the Python scripts developed for this study, including code for processing images, will be made publicly available at https://github.com/qwangmsk/Melanoma-Detect.
